# Efficient Cross-talk Reduction of Nanophotonic Circuits Enabled by Fabrication Friendly Periodic Silicon Strip Arrays

**DOI:** 10.1038/s41598-017-16096-9

**Published:** 2017-11-20

**Authors:** Yusheng Bian, Qiang Ren, Lei Kang, Yifeng Qin, Pingjuan L. Werner, Douglas H. Werner

**Affiliations:** 1 Computational Electromagnetics and Antennas Research Lab (CEARL), Department of Electrical Engineering, The Pennsylvania State University University Park, PA, 16802 USA; 2 School of Electronics and Information Engineering, Beihang University, Beijing, 100191 China

## Abstract

Reduction of the crosstalk between adjacent photonic components has been regarded as one of the most effective, yet most challenging approaches for increasing the packing density of photonic integrated circuits. Recently, extensive efforts have been devoted to this field, leading to a number of elaborate designs, such as waveguide supperlattice and nanophotonic cloaking, among others. Here we develop a simple and efficient crosstalk reduction approach for silicon-based nanophotonic circuits by introducing a periodic array of silicon strips between adjacent waveguides. Studies indicate that the coupling lengths can be extended by more than two orders of magnitude for a waveguide pair with an edge-to-edge distance of ~*λ*/3 at the telecommunication wavelength. Further investigations reveal that our method is effective for both strongly and weakly confined silicon photonic modes, and works well over a broad band of operational wavelengths. In addition, the crosstalk reduction technique is shown to be capable of improving the coupling lengths of other elements as well, such as vertical silicon slot waveguides. Our approach offers a promising platform for creating ultra-compact functional components that is fabrication friendly, thereby providing a feasible route toward the realization of photonic integrated circuits with ultra-high packing densities.

## Introduction

Silicon photonics has been identified as a key enabling technology that could provide a promising solution to the current bottleneck in conventional micro-electronics due to its unprecedented features including high bandwidth, low power consumption and reduced cost, as well as its full compatibility with complementary metal oxide semiconductor (CMOS) technology^1,2^. Owing to the large refractive index contrast between silicon and low-index materials (*e*.*g*. silica, air) and the resultant ability to enable extreme confinement of optical fields, silicon-on-insulator (SOI) offers a remarkable platform for building numerous ultra-compact, high-performance photonic components^3^. A wide variety of applications that are otherwise challenging to realize using conventional technologies, such as on-chip guiding, confining and processing of light signals, can be readily achieved in silicon-based nanophotonic chips and circuits^1^. Since silicon-based optical elements could share the same wafer and potentially provide significant process compatibility with current silicon micro-electronic devices, combining silicon photonics with microelectronics is believed to be the future path of integrated circuits (IC)^4^. However, compared to the state-of-the-art ICs, photonic integrated circuits (PICs) still suffer from considerably lower packing densities, which presents a challenging obstacle for further steps towards low-cost, large-scale, three-dimensional (3D) multilayer hybrid integrated circuits^5^.

Recently, a number of different approaches have been developed to further improve the integration density of PICs. One of the typical methods, among others, is replacing the traditional photonic devices with advanced nanophotonic components such as plasmonic configurations^6–10^ and metamaterial-based structures^11–14^ in order to further shrink the device footprint. Another potential solution that has attracted considerable research interest is to reduce the crosstalk between adjacent components, *i*.*e*. the leakage of light from one element to its neighbors^15^, so that the minimum spacing between individual devices can be decreased. Among the wide variety of crosstalk reduction strategies that have been under development in recent years, waveguide supper-lattices^16^ and nanophotonic cloaking^17^ represent highly efficient approaches capable of enabling half-wavelength-scale waveguide spacing with negligible crosstalk at telecommunication wavelengths, which is a significant improvement relative to the state-of-the-art technology. In these studies, elaborate design schemes based on the interlacing-recombination supercell principle or inverse-design techniques for discrete binary pixels were exploited in order to obtain optimized configurations. To further simplify the design process, here we develop an alternative and much simpler approach to reduce the crosstalk in nanophotonic circuits by introducing a periodic array of silicon strips between adjacent waveguides. Our design is straightforward, such that it neither requires a complex algorithm for structure optimization nor a sophisticated process in device fabrication. This approach can be applied to various types of photonic devices as well and enables efficient crosstalk reduction across a broad band of operation wavelengths.

## Results

The key to the control of crosstalk is to efficiently manage the spatial overlap between different modes and regulate the distribution of the evanescent waves in neighboring waveguides. As revealed in recent work^18,19^, the penetration depth of the evanescent fields into the surrounding medium is governed by the ratio of the permittivity components along different directions. This finding enables us to flexibly control the spatial field distribution of waveguides simply through tuning the permittivity ratios, leading to unprecedented confinement capabilities that are otherwise difficult to achieve using all-dielectric configurations^18^. Here we apply such a concept to reduce the overlap between the evanescent waves of the guided modes and minimize the crosstalk between neighboring silicon-based nanophotonic waveguides (see Methods). In our design, periodic silicon nano-strip arrays are introduced in between two neighboring silicon waveguides in order to adjust the penetration depth of the evanescent waves associated with the waveguide modes. It is worth noting that although similar configurations have been reported quite recently^20^, comprehensive studies regarding the effect of different geometries and their physical dimensions on the performance of crosstalk reduction are yet to be demonstrated. In addition, the effectiveness of the approach for weakly confined photonic modes and other types of silicon waveguides remains uncertain. Herein, by taking full consideration of various key factors such as waveguide type, geometric size, confinement capability and operating wavelength, we will carry out comprehensive studies to demonstrate the transformative potential of such an approach in crosstalk reduction.

**Figure 1:**
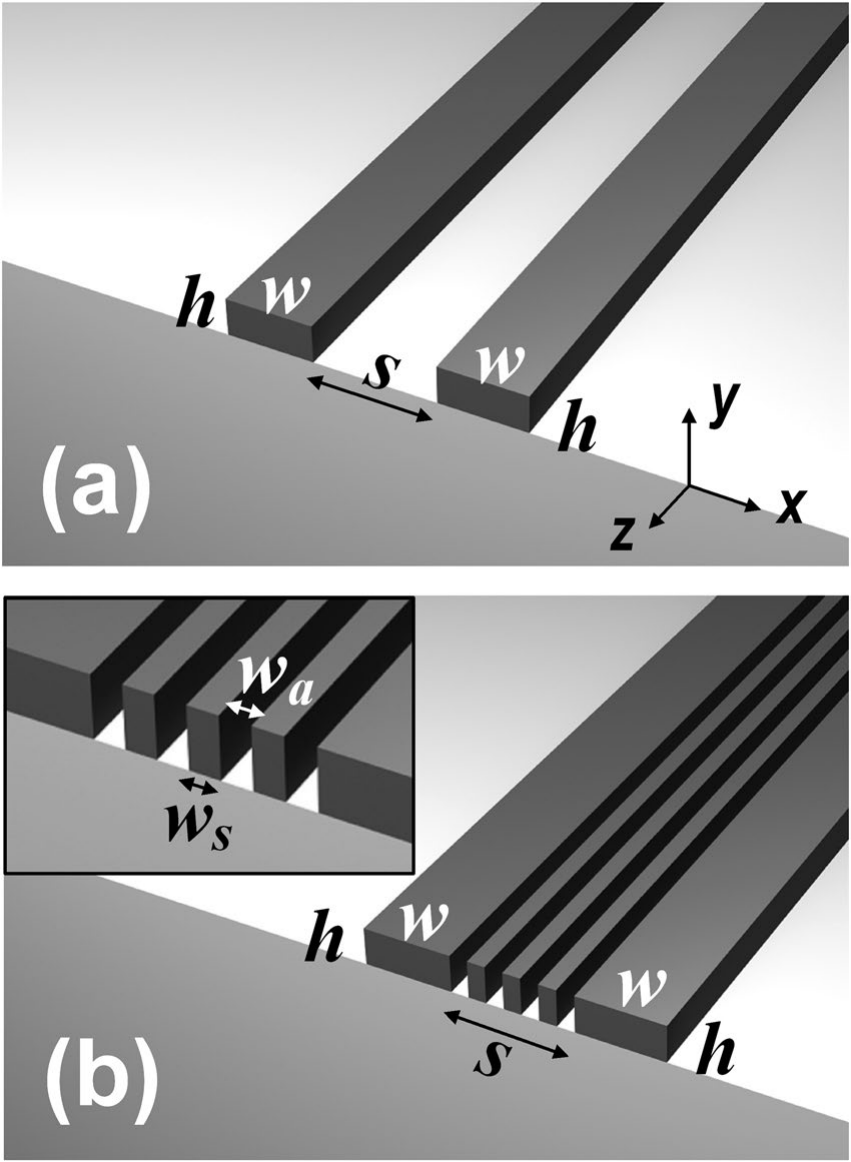
Schematics of (**a**) two identical silicon ridge waveguides (width *w* and height *h*) separated from each other with an edge-to-edge distance of *S*; and (**b**) the proposed configuration that comprises a periodic silicon strip array inserted in between the silicon waveguide pair, where the inset shows an enlarged view of the strip array. The substrates and claddings for the conventional and newly proposed configurations are silica and air, respectively. The widths of the silicon strip and the air slot are *w*_*s*_ and *w*_*a*_, respectively.

Figure 1(a) shows schematically the configuration of a coupling system that comprises two horizontally parallel silicon ridge waveguides with an edge-to-edge separation of *S*, whereas Fig. 1(b) illustrates the proposed configuration that incorporates an additional silicon strip array inserted between the two waveguides. Due to the periodic structure consisting of alternating silicon strips and air slots, the introduced configuration can be regarded as a highly anisotropic medium with a pronounced permittivity contrast along the horizontal and vertical directions. To quantitatively illustrate the effectiveness of the approach in reducing the waveguide crosstalk, we calculate the coupling lengths (*L*_*c*_) between two closely spaced waveguides with and without the silicon strip array. The coupling length is obtained based on the coupled mode theory (see Methods). The properties of the guided modes in the waveguide system are studied using a finite element method (FEM) based on the COMSOL Multiphysics software package. In the simulations, the air cladding and the silica substrate are modeled using refractive indices of 1 and 1.444, respectively. To represent the optical constant of silicon material, we use the experimentally measured refractive index data^21^, while an interpolation of the data was adopted for describing the wavelength dependence of the dielectric function. In the following, through a geometric optimization of the silicon strip array, we will show that the coupling length of the silicon ridge waveguides can be dramatically increased, which leads to a significantly reduced waveguide crosstalk.

**Figure 2:**
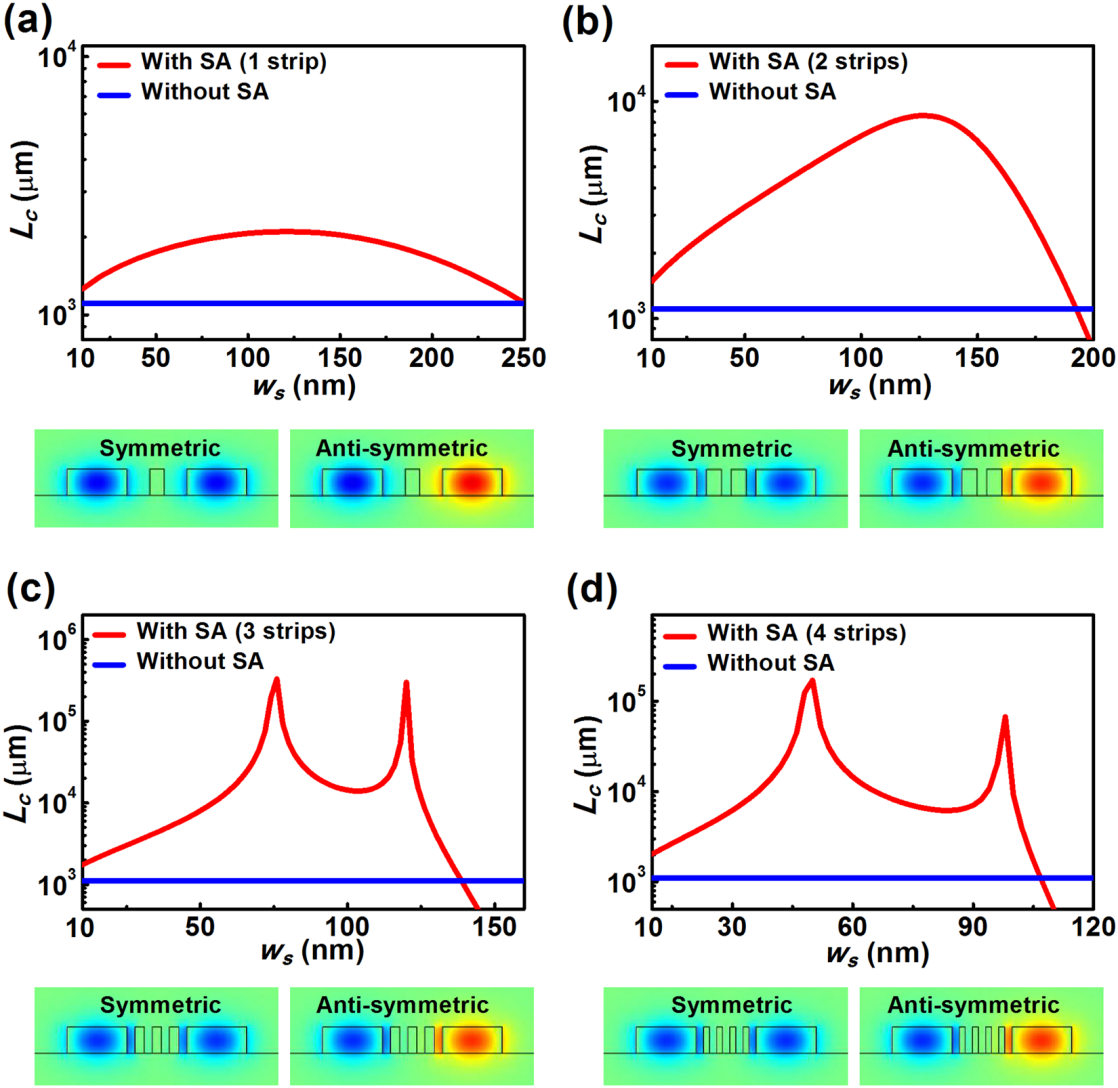
Effect of the strip width on the coupling length for configurations with and without the silicon strip array (SA). The widths of the silicon ridge waveguides are selected as 500 nm to ensure the existence of strongly confined TE modes. (**a**) A single strip; (**b**) two strips; (**c**) three strips; (**d**) four strips. Geometric parameters for the silicon waveguide pair are *w* = 500 nm, *h* = 220 nm, *S* = 500 nm. E_x_ field distributions of the symmetric and anti-symmetric super-TE-modes in the optimal coupling systems with the largest coupling length are plotted as well. The widths of the strip(s) corresponding to the optimal coupling lengths are *w*_*s*_ = 120 nm for a single strip (**a**), *w*_*s*_ = 126 nm for two strips (**b**), *w*_*s*_ = 76 nm for three strips (**c**) and *w*_*s*_ = 48 nm for four strips (**d**).

We start our investigation by considering the guided modes in configurations with 500-nm-wide silicon ridge waveguides, which are capable of supporting strongly confined TE photonic modes. Figure 2 shows the calculated coupling lengths for various strip widths in the coupling system and the corresponding distribution of the major electric field components for configurations achieving the optimal coupling lengths. To benchmark the performance of the proposed approach, the coupling length for the pure silicon waveguide pair without the silicon strip(s) is also plotted. As is clearly illustrated from Fig. 2(a–d), for all the considered cases, the introduction of the strip array can result in a dramatically extended coupling length relative to that of the pure silicon pair under the appropriate design scenarios. Near the optimal conditions, the coupling length is increased by a factor of 2 ~ 8 in a configuration with a single or two strips (Fig. 2(a,b)). While due to the potential of enabling a significantly larger range of permittivity ratios, considerably larger increases and more pronounced changes in the coupling length are observable for a silicon array having more than two strips, as seen in Fig. 2(c,d) and Table 1. Due to the fact that near-optimal permittivity ratios can be achieved by different silicon strip arrays with various combinations of the strip number and dimension, two local maxima in the coupling length curves are observed for the configurations with three and four silicon strips, as shown in Fig. 2(c,d). For all the considered cases, the corresponding field profiles of the symmetric and anti-symmetric super-modes at the optimal coupling lengths clearly indicate that the introduced silicon strip(s) are capable of effectively blocking the penetration of the silicon photonic modes into the surrounding medium. This results in weakened modal overlap between neighboring structures, and consequently, enhanced coupling lengths as well as reduced waveguide crosstalk. As shown in Table 1, the largest coupling length achieved by our proposed configuration under the considered circumstances is more than two orders of magnitude greater than that obtained in a conventional silicon pair, suggesting a significant reduction in crosstalk for closely spaced structures. We also note that the width of the silicon strip corresponding to such an optimal coupling length is 76 nm, which results in an aspect ratio ~ 1:3, making the configuration feasible to manufacture using standard nanofabrication techniques. For example, electron beam lithography defining the pattern of the periodic strips, followed by inductive coupled plasma etching could be employed^20,22^. It is also worth noting that for relatively wide silicon strips, the air gaps between the strips and those between the strips and the ridge are relatively small, which significantly weakens the ‘blocking effect’. This leads to increased overlap of the evanescent fields, and thus significantly reduced coupling lengths (see, *e*.*g*., w_s_ = 200 nm in Fig. 2(b)).

**Table 1:** Optimal coupling lengths for different configurations supporting strongly confined silicon photonic modes at 1.55 μm (*w* = *S* = 500 nm).

Configuration (*λ* = 1.55 μm)	Optimal *L*_*c*_ (μm)
Silicon pair without array	1.1 × 10^3^
Silicon pair with a single strip	2.1 × 10^3^
Silicon pair with two strips	8.5 × 10^3^
Silicon pair with three strips	3.32 × 10^5^
Silicon pair with four strips	1.71 × 10^5^
Silicon pair with five strips	1.07 × 10^5^

**Figure 3:**
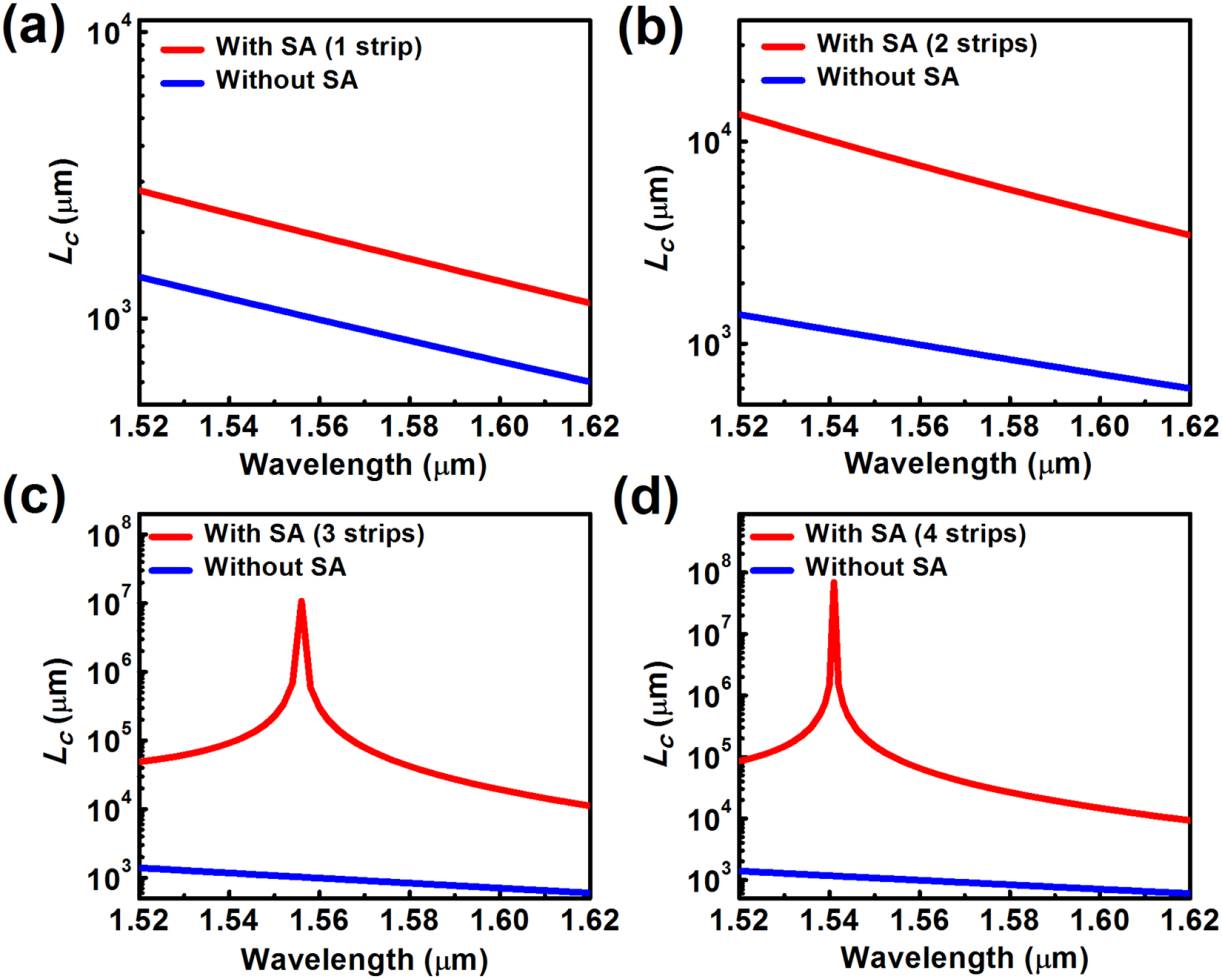
Dependence of the coupling length on the working wavelength for configurations with and without (**a**) a single silicon strip (*w*_*s*_ = 120 nm); (**b**) two silicon strips (*w*_*s*_ = 126 nm); (**c**) three silicon strips (*w*_*s*_ = 76 nm); (**d**) four silicon strips (*w*_*s*_ = 48 nm). Other structural parameters are *w* = 500 nm, *h* = 220 nm and *S* = 500 nm.

In addition, our further calculations indicate that the crosstalk reduction approach also works well over a broad range of wavelengths, as revealed in Fig. 3. For the single and two strip cases, the coupling length is increased by a factor of 2 ~ 8 within 1.52 ~ 1.62 μm. Although monotonic trends are seen for the considered wavelength range, non-monotonic behaviors with local optimal coupling lengths are observable for wavelengths shorter than 1.52 µm (See Supplymentary Information). While for structures with three or four strips, the coupling length can be extended by 1 ~ 4 orders of magnitude within the considered wavelength range, which is nearly 100 meters under the optimized condition (Fig. 3(d)). Such an extremely large coupling length enables the silicon ridge waveguides to be packaged in close proximity to each other, which is highly desirable for creating ultra-compact photonic components and ultra-high-density PICs. It is worth mentioning that although the linewidth is relatively narrow regarding the optimal coupling efficiency for the three and four strip cases, the coupling lengths near the optimal position are extremely large, which is a 3 ~ 4 orders of magnitude improvement relative to the configuration without the silicon strip array. Therefore, even if there is a wavelength shift, greatly extended coupling length and significantly reduced crosstalk can still be realized. On the other hand, a potential way to further increase the operating linewidth is to incorporate silicon strips with different widths (such as apodized configurations), which can be designed based on advanced global optimization algorithms and will be included in our future work.

**Figure 4:**
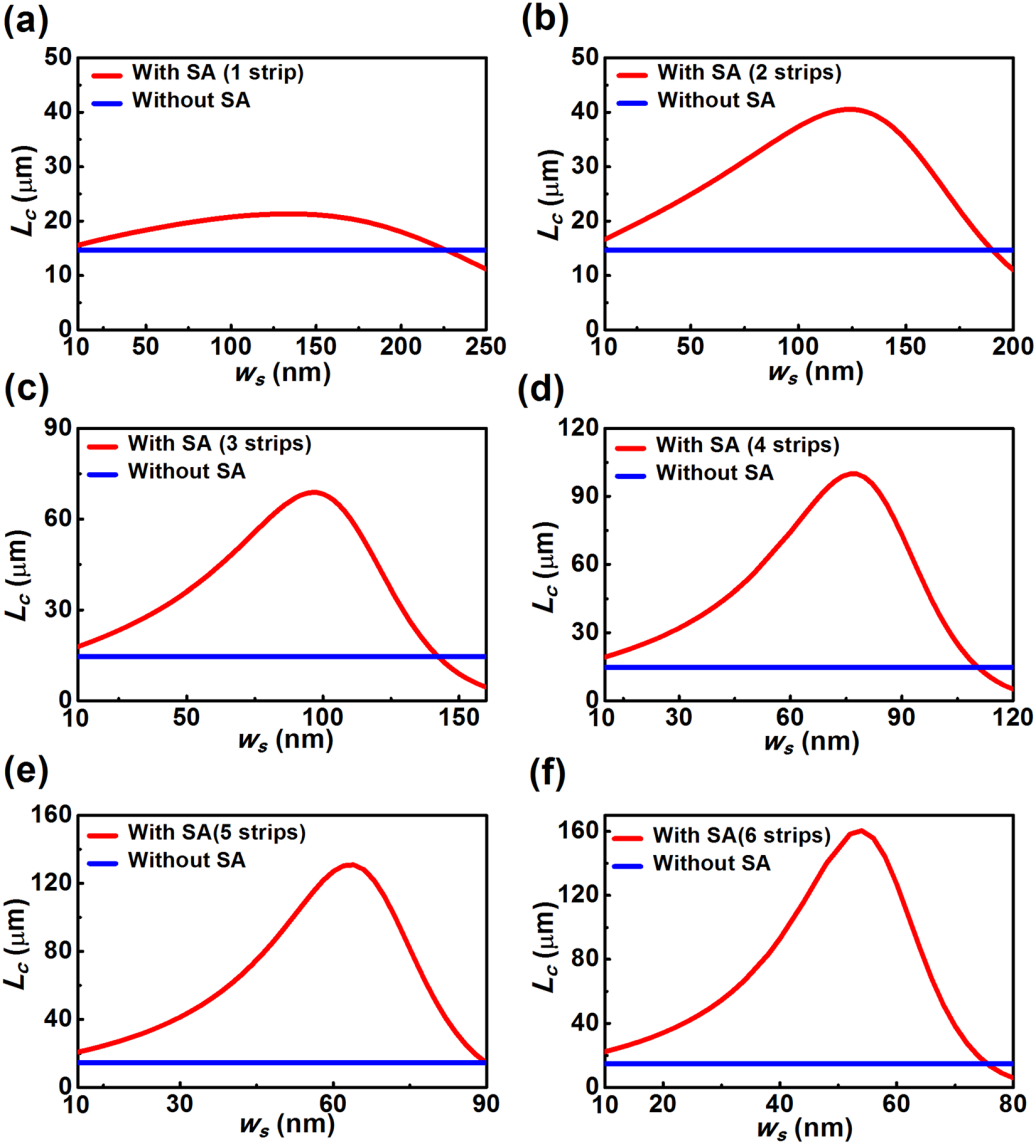
Dependence of the coupling length on the width of the silicon strip for configurations with and without the silicon strip array (SA). The widths of the considered silicon ridge waveguides are 300 nm, which support weakly confined TE photonic modes. (**a**) A single strip; (**b**) two strips; (**c**) three strips; (**d**) four strips; (**e**) five strips; (**f**) six strips. Parameters are *w* = 300 nm, *h* = 220 nm, *S* = 500 nm. Here, the widths of the strip(s) with optimal coupling length are *w*_*s*_ = 130 nm for a single strip (**a**), *w*_*s*_ = 124 nm for two strips (**b**), *w*_*s*_ = 96 nm for three strips (**c**), *w*_*s*_ = 76 nm for four strips (**d**), *w*_*s*_ = 64 nm for five strips (**d**) and *w*_*s*_ = 54 nm for six strips (**e**).

In addition to the above studies concerning highly confined silicon modes, here we conduct further investigations on the crosstalk reduction for configurations supporting weakly confined dielectric modes. In the study, the width of the silicon ridge waveguide is selected as 300 nm and the effect of the number and size of the silicon strips on the coupling length is analyzed in detail, as shown in Fig. 4 and Table 2. Clearly, the introduction of the silicon strip(s) results in a notable enhancement of the coupling length within a wide parameter range for all the considered cases, which further confirms the effectiveness of the approach in crosstalk reduction for relatively narrow silicon waveguides. As seen from Fig. 4(c) and Table 2, the optimal coupling length for the three-strip-case is nearly four times larger than that of the pure waveguide pair. It is worth noting that the coupling lengths are dependent not only on the geometry and dimension of the silicon strips, but also on the field confinement and evanescent waves associated with the silicon waveguides. Since the 300 nm-wide silicon waveguide exhibits much weaker confinement as compared to the 500 nm case, the improvement in the coupling lengths is limited, and less sensitive behavior can be observed with the dimension change of the silicon strip array.

**Table 2:** Optimal coupling length for different configurations supporting weakly confined silicon photonic modes at 1.55 μm (*w* = 300 nm, *S* = 500 nm).

Configuration (*λ* = 1.55 μm)	Optimal *L*_*c*_ (μm)
Silicon pair without array	14.6
Silicon pair with a single strip	21.3
Silicon pair with two strips	40.6
Silicon pair with three strips	68.8
Silicon pair with four strips	99.9
Silicon pair with five strips	130.9
Silicon pair with six strips	160.4

**Figure 5:**
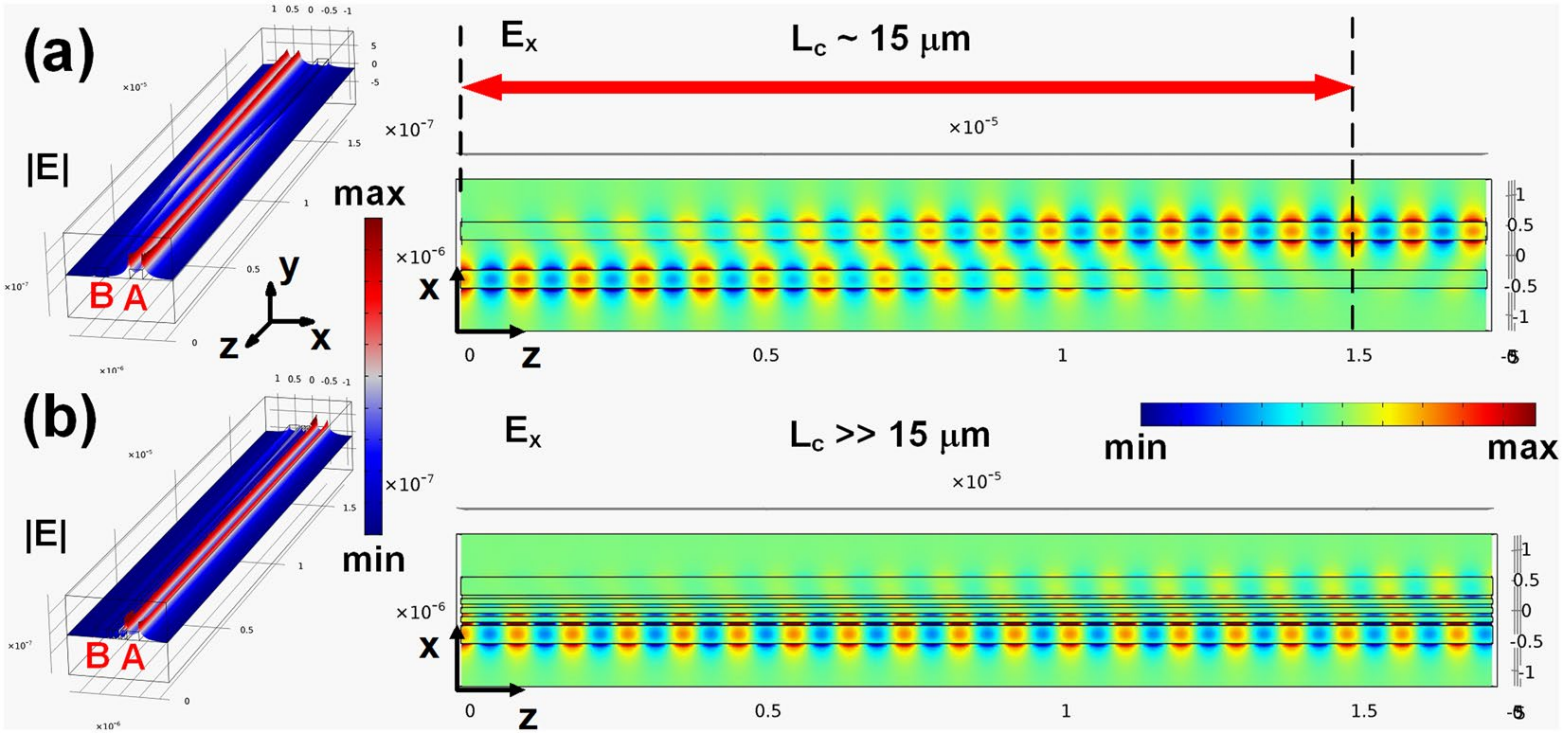
(**a**,**b**) 3D full wave simulation of the coupling between silicon waveguides without and with the strip array (three strips). For both configurations, the lengths of the waveguide pair and the strip array are 17 μm. Other structural parameters for the cross-section of the configurations are: *w* = 300 nm, *h* = 220 nm, *S* = 500 nm, *w*_*s*_ = 96 nm and *w*_*a*_ = 53 nm. The left images show the 3D normalized electric field profiles, whereas the right figures demonstrate the corresponding 2D transmitted E_x_ field distributions in the *x*-*z* plane through the center of the configurations.

3D full-wave simulations using COMSOL Multiphysics allow us to gain deeper insight into the mode coupling from one waveguide to the other, which further validates the 2D simulation results based on coupled mode theory. As shown in Fig. 5, the estimated coupling lengths are 15 μm and ≫15 μm for the configurations without and with the three silicon strips, respectively, which agrees quite well with the results shown in Table 2. Our calculations further reveal that the crosstalk between the two neighboring silicon waveguides has been reduced down below −8 dB for the specific configuration. Here it is worth mentioning that by introducing even more strips, the optimal coupling length can be further increased and the crosstalk value can be reduced as well. For example, by exploiting six strips, the maximal coupling length can be more than one order of magnitude larger than that achieved by the conventional structure, as illustrated in Table 2. Our studies also indicate that the proposed crosstalk reduction approach is robust and exhibits good tolerance to possible fabrication imperfections, such as the width variation in the silicon strips (see Supplementary Information for details).

**Figure 6:**
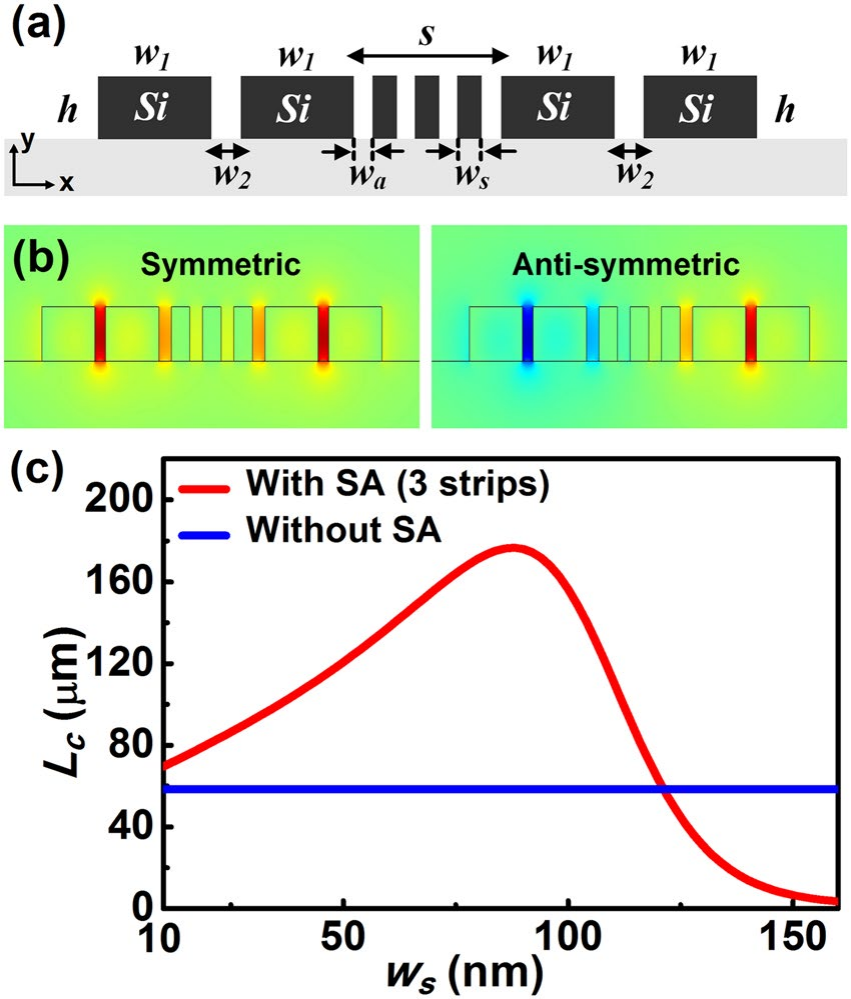
Crosstalk reduction for silicon slot waveguides. (**a**) Sketch of the proposed configuration consisting of a periodic silicon strip array inserted in between the silicon slot waveguide pair; (**b**) E_x_ field distributions of the symmetric and anti-symmetric super-TE-modes in a typical coupling system enabling the optimal coupling length; (**c**) effect of strip width on the coupling length for configurations with and without the three-strip array (SA). Parameters are *w*_*1*_ = 250 nm, *w*_*2*_ = 50 nm, *h* = 220 nm, *S* = 500 nm. The widths of the strip and the air gap at the maximum coupling length are *w*_*s*_ = 88 nm and *w*_*s*_ = 59 nm, respectively. The field profiles illustrate relatively weak modal overlap between neighboring structures at the optimal coupling length.

Besides the standard silicon ridge waveguide discussed above, the crosstalk reduction approach is also effective for enhancing the coupling lengths between other typical silicon-based photonic waveguides, such as silicon slot structures^22–24^. Owing to the remarkable capability of providing strong optical confinement and local field enhancement inside the nanoscale low-index slot region, slot waveguides enable the realization of various compact photonic components^25–28^ as well as facilitate numerous applications^29–31^. Reduction of the crosstalk between adjacent slot waveguides is therefore of great importance for the miniaturization of integrated photonic components and circuits comprised of silicon slot structures. Figure 6 shows the schematic of the configuration that incorporates additional silicon strips between the two neighboring silicon slot waveguides, as well as the simulation results of the coupling lengths. Similar to that observed for the silicon ridge waveguide case, the coupling lengths can be dramatically extended through carefully engineering the size of the silicon strip array. For the three-strip-case considered in our study, an optimal coupling length can be realized when the strip width reaches 88 nm, which is 3 times as large as that of the traditional silicon slot waveguide pair without the strip array. By further adjusting the physical dimension of the slot waveguide, more tightly confined modes can be supported, which could potentially lead to even more pronounced enhancement in the coupling length, and thus dramatically reduce waveguide crosstalk even further. It is also worth mentioning that due to the significantly weakened ‘blocking’ effect for small air slots, the coupling length decreases more dramatically when the strip size is relatively large, similar to that observed in silicon ridge waveguides (Figs. 2 and 4).

## Discussion

It is worth mentioning that the silicon strips introduced in our studies are exclusively based on periodic configurations with exactly the same width and air gap size. To find an even more feasible and efficient crosstalk-reduction approach, more research work is required. This is particularly true for the weakly confined mode case as discussed above. It is found that an enhanced optimal coupling length can be expected through increasing the number of the silicon strips. However, the increasingly narrower silicon strips in these scenarios would add additional complexity to the device fabrication. To overcome such a limitation, one promising approach might be to introduce a non-periodic silicon strip array (instead of the periodic arrays studied here), along with a powerful global optimization technique such as the genetic algorithm^32–34^ to determine the best spacing between strips. Detailed discussions relating to these alternative crosstalk reduction strategies, as well as design considerations for densely packed devices (*e*.*g*. the waveguide spacing is less than 0.3 *λ*) and partially etched silicon waveguides (widely exploited for active devices such as electro-optic modulators) will be reported in our future work.

In conclusion, an efficient approach for reducing the crosstalk between adjacent silicon-based nanophotonic waveguides has been developed, which is realized by introducing an array of silicon strips between neighboring waveguides. Studies show that significantly extended coupling lengths across a broad band of operating wavelengths are achievable under appropriate design conditions. Our approach is applicable to both strongly and weakly confined photonic modes, and works effectively for other types of silicon-based components as well, including silicon slot waveguides. The crosstalk reduction technique offers a simple and fabrication friendly solution to miniaturizing the footprint of photonic components and paves the way for the realization of ultra-high-density PICs.

## Methods

The characteristics of the guided modes in conventional coupled silicon ridge/slot waveguides and the proposed silicon ridge/slot-based waveguide systems incorporating periodic silicon strips were investigated numerically by solving the Helmholtz equation using the eigenmode solver of the finite element method (FEM) based software COMSOL Multiphysics. A scattering boundary condition is applied to mimic the open boundary. Convergence tests ensure that the numerical boundaries and meshing did not interfere with the solutions. The crosstalk between adjacent silicon-based waveguides is evaluated by calculating the coupling lengths, which can be obtained based on the coupled mode theory^35^:

1 $${L}_{c}=\pi /|{k}_{s}-{k}_{a}|$$

where *k*_*s*_ and *k*_*a*_ are the wavenumbers of the symmetric and anti-symmetric modes of two coupled waveguides, respectively. Substituting ${k}_{s}={k}_{0}{n}_{s}=2\pi {n}_{s}/\lambda$ and ${k}_{a}={k}_{0}{n}_{a}=2\pi {n}_{a}/\lambda$ into Eq. (1), it follows that:

2 $${L}_{c}=\lambda /(2|{n}_{s}-{n}_{a}|)$$

where *n*_*s*_ and *n*_*a*_ are the real parts of the modal effective indexes for the symmetric and anti-symmetric modes, respectively.

The mechanism of our crosstalk reduction approach is the same as that reported in ref.^17^ and ref.^18^. The coupling between two adjacent silicon ridge/slot waveguides is highly dependent on the evanescent waves. By tuning the penetration depth (skin depth) of the evanescent wave into the surrounding medium, the coupling between these waveguides can be well controlled.

The dispersion relation for light waves penetrating from the first medium (*e*.*g*. silicon) into the second medium (*e*.*g*. air) is,

3 $$\frac{{({k}_{x}^{||})}^{2}}{{{\rm{\varepsilon }}}_{y}}+\frac{{({k}_{y}^{\perp })}^{2}}{{{\rm{\varepsilon }}}_{x}}={({k}_{0})}^{2}$$

In Equation (3), ${k}_{x}^{||}$ and ${k}_{y}^{\perp }$ are the parallel and perpendicular components of the wave vector in the second medium, respectively, whereas *ε*_*x*_ and *ε*_*y*_ are the dielectric constants of the second medium parallel and perpendicular to the interface. *k*_0_ is the wave vector in free space. Based on Equation (3), we are able to obtain an expression for the evanescent wave decay constant in the second medium:

4 $${k}_{y}^{\perp }=\sqrt{\frac{{\varepsilon }_{x}}{{\varepsilon }_{y}}}\sqrt{{\varepsilon }_{y}{({k}_{0})}^{2}-{({k}_{x}^{||})}^{2}}$$

From Equation (4), one can see that the penetration depth of the evanescent fields into the second medium is governed by the ratio of the permittivity components. By engineering the ratio of the permittivity parallel to the interface to the one that is perpendicular to the interface, we are able to further control the decay rate of the evanescent waves for the silicon ridge and slot waveguides. Therefore, through changing the dimensions of the silicon strip array (treated as an anisotropic medium), it is possible to control the permittivity along the horizontal and the vertical directions. This allows further modifications in the penetration depth of the evanescent waves and the coupling lengths between waveguides.

## Electronic supplementary material

Supplementary Information
