# Supplementary material for: Efficient Cross-talk Reduction of Nanophotonic Circuits Enabled by Fabrication Friendly Periodic Silicon Strip Arrays
Source: Sci Rep. 2017 Nov 20;7:15827. doi: 10.1038/s41598-017-16096-9 (PMC5696539; doi:10.1038/s41598-017-16096-9)
Supplement: Supplementary file 1 — Supplementary Information [file 41598_2017_16096_MOESM1_ESM.pdf]

Supplementary Information for:

Efficient Cross-talk Reduction of Nanophotonic Circuits Enabled by Fabrication Friendly Periodic Silicon Strip Arrays

Yusheng Bian[1,†], Qiang Ren[2,†], Lei Kang[1], Yifeng Qin[1], Pingjuan L. Werner[1] and Douglas H. Werner[1,*]

[1]*Computational Electromagnetics and Antennas Research Lab (CEARL)*

Department of Electrical Engineering, The Pennsylvania State University

University Park, PA, 16802, USA

[2]*School of Electronics and Information Engineering, Beihang University, Beijing 100191, China*

** Corresponding Author. Email: dhw@psu.edu*

† These authors contributed equally to this work

S1. Crosstalk analysis for configurations with fabrication imperfections

In the fabrication process of the proposed configurations incorporating silicon strip arrays, accurate control of the strip width may present a challenge. Here we conduct further studies on the tolerance of the coupling length and crosstalk against such a fabrication imperfection. Supplementary Fig. 1 shows the dependence of the coupling length on the operating wavelength for different strip arrays. Variations in the strip array width of +/-5 nm are considered in all cases to account for the potential fabrication error. It is clearly shown that for the single and two strip cases, the coupling lengths remain almost unchanged despite the variation in the strip width. While for configurations with three or four strips, although blue- and red-shifts in the

spectrum are observable for narrower (-5 nm variation) and wider (+5 nm variation) strips, the coupling length can still be extended by 1 ~ 3 orders of magnitude as compared to the configuration without the silicon strip array. In addition, for waveguides with width variation of the strips along the propagation direction, increased coupling length and reduced crosstalk can still be realized with a certain tolerance range as compared to the waveguides without any silicon strips. These results further verify the robustness of the proposed crosstalk reduction approach, which works well over a broad range of wavelengths for configurations with realistic fabrication imperfections.

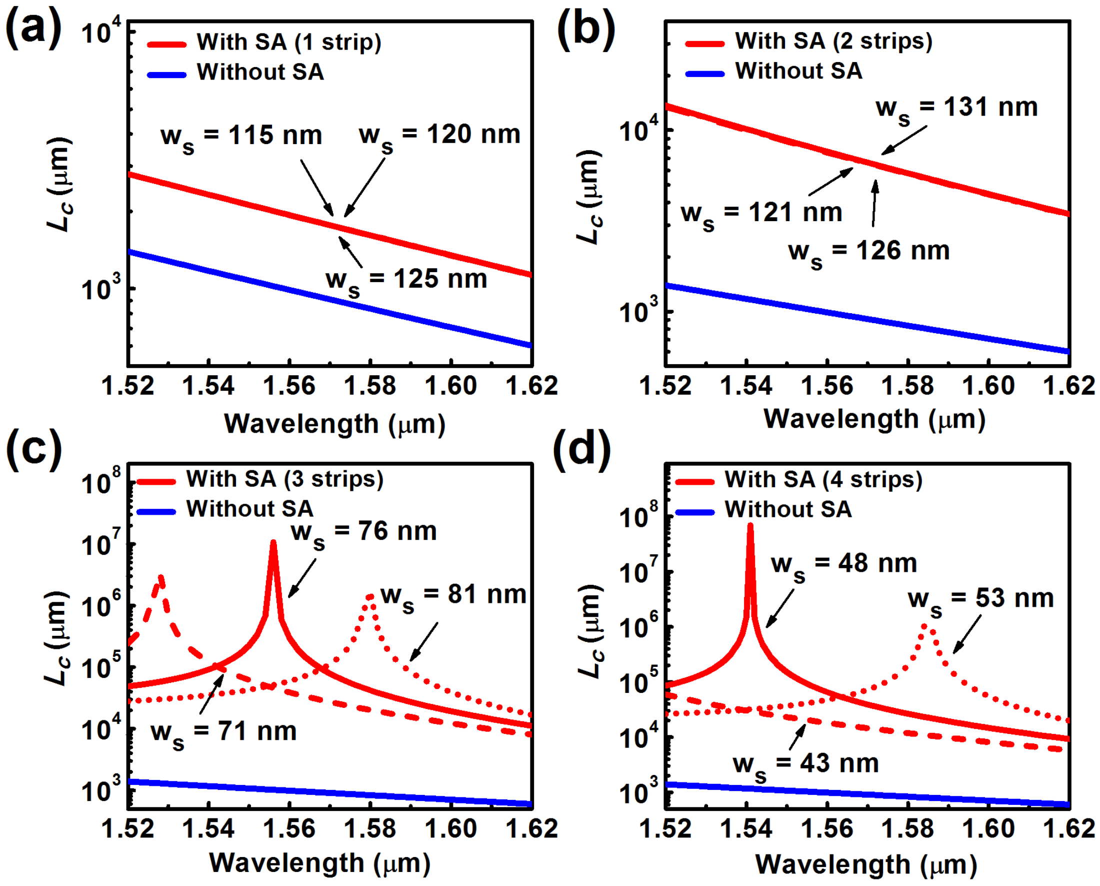

Supplementary Figure 1 Dependence of the coupling length on the working wavelength for configurations with and without (a) a single silicon strip (w_s = 115 nm, w_s = 120 nm, w_s = 125 nm); (b) two silicon strips (w_s = 121 nm, w_s = 126 nm, w_s = 131 nm); (c) three silicon strips (w_s = 71 nm, w_s = 76 nm, w_s = 81 nm); and (d) four silicon strips (w_s = 43 nm, w_s = 48 nm, w_s = 53 nm). Other structural parameters are w = 500 nm, h = 220 nm and S = 500 nm. Variations in the silicon strip width of +/- 5

nm are considered in this study. Due to the slight changes in the coupling lengths for the single strip and two strips cases, the curves are overlapping in (a) and (b).

S2. Crosstalk analysis within the wavelength range of 1.26 ~ 1.62 μm

We also observed non-monotonic behavior for the one- and two-strip cases when the wavelength is shorter than 1.52 μm. Taking the two-strip case as an example, an optimal coupling efficiency with a peak in the spectrum appears at ~ 1.345 μm, as shown in Supplementary Fig. 2. While for the one-strip case, the optimal coupling efficiency is observed at wavelengths below 1.26 μm, which is not plotted here since it is outside of the wavelength range of interest.

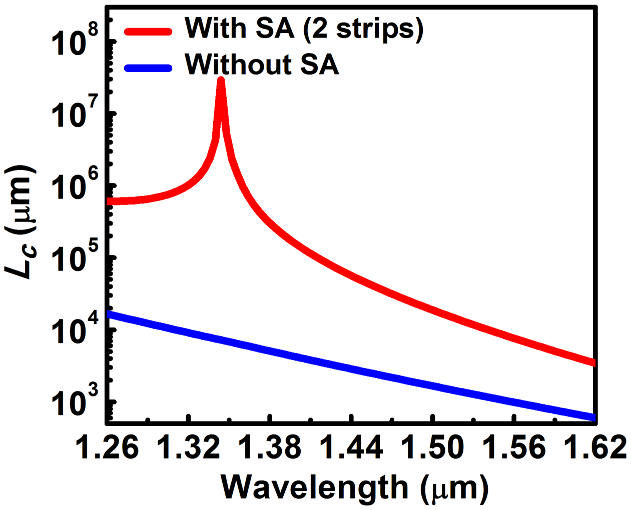

Supplementary Figure 2 Dependence of the coupling length on the working wavelength for configurations with and without two silicon strips (w_s = 126 nm). Other structural parameters are w = 500 nm, h = 220 nm and S = 500 nm. A significantly larger wavelength range is considered, which reveals the optimal coupling length at ~1.345 μm.
